# Does knee awareness differ between different knee arthroplasty prostheses? A matched, case-control, cross-sectional study

**DOI:** 10.1186/s12891-016-1001-3

**Published:** 2016-04-01

**Authors:** Morten G. Thomsen, Roshan Latifi, Thomas Kallemose, Henrik Husted, Anders Troelsen

**Affiliations:** Department of Orthopaedic Surgery, Copenhagen University Hospital Hvidovre, Kettegård Allé 30, 2650 Hvidovre, Denmark

**Keywords:** Knee, Arthroplasty, TKA, Rehabilitation, Awareness, FJS, OKS, Design

## Abstract

**Background:**

Low knee awareness after Total Knee Arthroplasty (TKA) has become the ultimate goal in trying to achieve a natural feeling knee that meet patient expectations. To accommodate this manufacturers of TKAs have developed new prosthetic designs that potentially could give patients a more natural feeling knee during activities.

The purpose af this study was to compare the Forgotten Joint Score (FJS) and Oxford Knee Score (OKS) of patients treated with a previous generation standard Cruciate Retaining (CR) TKA to the scores obtained by patients treated with a newer generation CR TKA or a mobile bearing CR TKA.

**Methods:**

We identified all patients receiving a new generation CR TKA or mobile bearing TKA at our institution between 2010 and 2012. These were matched to a population of patients receiving a standard CR TKA regarding age, gender, year of surgery, Kellgren-Lawrence (KL) grade and pre- and postoperative knee alignment. Patients were asked to complete the FJS and OKS questionnaires. Of the 316 patients completing the survey 64 standard CR TKAs to 35 new generation CR TKAs and 121 standard CR TKAs to 68 mobile bearing TKAs were matched. The FJS and OKS scores of the three TKA designs were compared.

**Results:**

When comparing the new generation CR TKAs to the standard CR TKAs we found statistically significant higher OKS and FJS scores (6 (*p* = 0.04) and 16 (*p* = 0.03) points respectively) for the new generation CR TKAs. When comparing the mobile bearing TKAs to the standard CR TKAs we found a statistically significant higher OKS score (3 points, *p* = 0.04), and a higher but non-significant FJS score (4 points, *p* = 0.48) for the mobile bearing TKAs.

**Conclusions:**

Patients receiving the new generation CR TKA obtained higher FJS and OKS scores when compared to patients receiving a standard CR TKA, indicating that the use of this newer prosthetic design facilitate less knee awareness and better function after TKA.

## Background

New prosthetic designs have been developed with the aim of giving patients enhanced knee function and a more natural feeling knee during activities of daily living (ADL). Examples of these new prosthetic designs are High-Flex-, Mobile Bearing- and gender specific TKA’s. Earlier studies investigating the outcome after treatment with these new prosthetic designs using conventional outcome measures (e.g. pain, ROM, revision rates and functional Patient Reported Outcome Measures (PROM’s)) have, however, not been able to show clinical benefits when compared to previous generation knee prosthetic designs [1–6].

It can be argued that these new prosthetic designs may result in clinical benefits that have not been captured by conventional outcome measures. In order to evaluate how natural the knee feels after TKA, a new scoring system, the Forgotten Joint Score (FJS), has recently been developed [7]. The FJS questionnaire is a 12-item scoring system based on the patients’ awareness of an artificial joint during ADL. In other terms, knee awareness describes the patients’ ability to forget the artificial joint in everyday life, which is considered to be the ultimate goal after total knee arthroplasty. The FJS questionnaire has shown promising results in earlier studies [7–9].

The purpose of this study was to investigate if patients treated with a newer generation cruciate retaining (CR) TKA or a mobile bearing (MB) CR TKA will have less knee awareness and greater knee function when compared to patients treated with a previous generation standard CR TKA.

## Methods

In this matched, case-control cross-sectional study we identified all patients receiving a primary unilateral cemented newer generation fixed bearing CR TKA (Vanguard CR, Biomet, Warsaw, Indiana) or an uncemented mobile Bearing (MB) CR TKA (Vanguard ROCC, Biomet, Warsaw, Indiana) at our institution (Copenhagen University Hospital Hvidovre, Denmark) between January 2010 and January 2013. Patients were identified through local database search.

The Vanguard CR prosthesis is a newer generation cruciate retaining, fixed bearing TKA. The femoral component has a deeper, longer trochlear groove in order to facilitate patellar tracking in all degrees of knee ROM. The anterior flange is narrower to avoid overstuffing of the anterior compartment and the femoro-tibial articulate surface is designed with an increased contact area during high degrees of flexion in order to increase stability. The Vanguard ROCC MB-CR TKA was developed with the intent of allowing tibio-femoral rotation during flexion in order to mimic the kinematics of the natural knee during full ROM, thereby in theory increasing knee function and patient satisfaction during ADL. The femoral component of the Vanguard ROCC prosthesis also has a deeper, longer trochlear groove in order to optimize patellar tracking in all degrees of ROM. Both prosthetic designs have shown good clinical results in earlier studies [10–13].

**Fig. 1:**
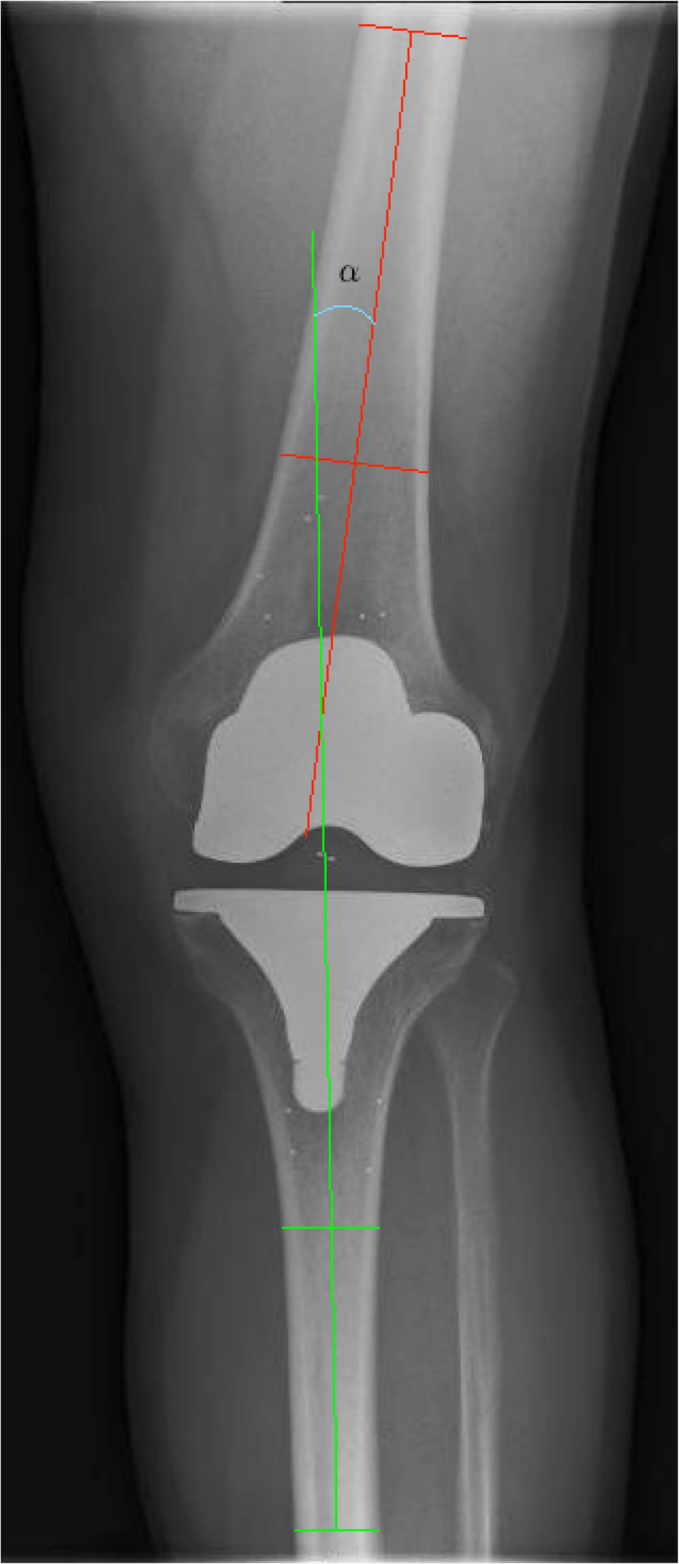
Measurement of the anatomical alignment (α) of a knee after primary TKA with a Vanguard ROCC knee prosthesis as presented by Petersen et al

Patients who had undergone prior knee surgery or underwent revision surgery (1 newer generation CR, 2 MB-CR and 11 previous generation CR) were excluded leaving a primary study cohort of 48 newer generation CR and 117 MB-CR TKA’s. For all patients, gender, year of surgery and age at time of surgery was documented. Preoperative radiographs of all knees were evaluated with regard to degree of osteoarthritis using the Kellgren-Lawrence (KL) grading scale, which has previously been validated and has been proven to be highly reproducible when used in the grading of knee osteoarthritis [14, 15]. Pre- and postoperative antero-posterior (AP) knee anatomical alignment were measured on pre- and postoperative radiographs for all knees according to the method presented by Petersen et al. [16] (Fig. 1).

Each group of TKA’s were then matched 1:2 to a population of patients receiving a previous generation primary cemented CR TKA (AGC, Biomet, Warsaw, Indiana) at our institution during the same period of time regarding age at time of surgery, gender, year of surgery, KL-grade and pre- and postoperative anatomical knee alignment. The AGC Prosthesis is a widely used TKA system which has shown good clinical results and long term survival in earlier studies [5, 17–20].

When matching was performed, a study cohort consisting of 360 knees (41 newer generation CR to 82 previous generation CR TKA’s and 79 MB-CR to 158 previous generation CR TKA’s) was found to be eligible for participation. All patients were operated in a fast-track setting using a standard medial para-patellar approach, establishment of bony cuts and knee balance using a measured resection technique, and they followed the same standardized postoperative rehabilitation program [21]. Five senior surgeons dedicated to TKA surgery performed all procedures.

In January 2014, all patients were invited to participate in this study giving a follow-up period of 1–4 years. Each patient received a set of questionnaires consisting of a Danish version of the FJS- and Oxford Knee Score (OKS) questionnaires. The OKS is a scoring system developed in order to evaluate the outcome after TKA based on patient reported outcome. The OKS 12-item questionnaire has previously been validated [22]. Earlier studies have found that a difference in OKS score of 4 to 5 points can be defined as a minimal clinically important difference (MCID) [23].

Three hundred thirty-one patients (38 newer generation CR, 78 MB-CR, 215 previous generation CR TKA’s) completed the questionnaires sufficiently resulting in a response completeness of 85.4 %. The knees were then re-matched 1:2 when possible, otherwise 1:1, leaving 35 newer generation CR to 64 previous generation CR TKA’s and 68 MB-CR to 121 previous generation CR TKA’s eligible for analysis. The demographics of patients included in the analysis after re-matching is presented in Table 1. There were no statistically significant differences in any of the demographic parameters when the matched groups were compared.

**Table 1:** Demographics of patients included in the analysis after re-matching

		Newer generation CR vs previous generation CR		MB-CR vs previous generation CR	
		(*n* = 35)	(*n* = 66)	(*n* = 68)	(*n* = 121)
Age		63 (11)	67 (10)	62 (8)	65 (8)
Gender	Male	14 (40 %)	28 (44 %)	27 (40 %)	48 (40 %)
	Female	21 (60 %)	36 (56 %)	41 (60 %)	73 (60 %)
Year of surgery	2010	0	0	9 (13 %)	16 (13 %)
	2011	3 (9 %)	5 (8 %)	33 (49 %)	58 (48 %)
	2012	28 (80 %)	51 (80 %)	26 (38 %	47 (39 %)
	2013	4 (11 %)	8 (12 %)	0	0
KL-grade	1-2	13 (37 %)	25 (39 %)	26 (38 %)	47 (39 %)
	3-4	22 (63 %)	39 (61 %)	42 (62 %)	74 (61 %)
Preop. axis		1° (5°)	1° (6°)	1° (5°)	1° (5°)
Postop. axis		4° (3°)	5° (3°)	4° (3°)	5° (3°)

Numbers presented in brackets are standard deviations (SD) where data is normally distributed or percentages of all

**Fig. 2:**
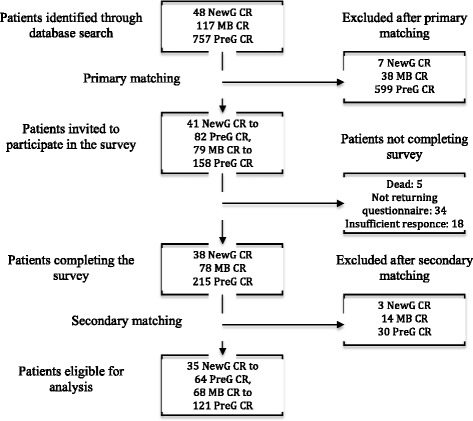
Flow diagram describing patients invited to participate in the study and included in the analysis

The FJS and OKS scores were then calculated and compared between the matched groups. The range for the total FJS-score is 0–100, with 100 being the best possible score. The range of the total OKS-score is 0–48, with 48 being the best possible score. A flow diagram for the current investigation is found in Fig. 2.

### Statistics

Comparison of the FJS score was done using weighted one-sample *t*-test, weights were assigned based a 1:1 or 1:2 matching. We evaluated the difference of the FJS between each matched pair, in the cases of a 1:2 matching the mean of the two scores was used. This creates unequal variance within the differences, to adjust for this, weights of √1/2 for 1:1 cases and √2/3 for 1:2 cases were assigned. The same method was used in the analysis of the OKS. A *p*-value of less than 0.05 was considered statistically significant, all matching and analysis was done using R 3.0.2 (R foundation for Statistical Computing, Vienna, Austria).

## Results

### Forgotten joint score

When comparing the newer generation CR and previous generation CR TKA knees, we found a statistically significant higher FJS score for the newer generation CR group by 15 points (*p* = 0.033) (Table 2). The newer generation CR prosthesis achieved statistically significant higher scores in FJS questions 1, 4, 5, 6 and 8 (Table 3), when compared to the previous generation CR TKA. When comparing the FJS scores of the MB-CR and previous generation CR TKA group, we found a higher FJS score for the MB-CR knees by 5 points. This however was not statistically significant (*p* = 0.49).

**Table 2:** FJS and OKS scores (mean) of the matched groups

	Newer generation CR vs Previous generation CR		*p*-value
	(*n* = 35)	(*n* = 64)	
FJS	59 (27)	44 (28)	0.033
OKS	37 (11)	32 (11)	0.039
	Mobile Bearing CR vs. Previous generation CR		
	(*n* = 68)	(*n* = 121)	
FJS	57 (28)	52 (30)	0.49
OKS	38 (9)	34 (11)	0.047

Standard deviations are presented in brackets

**Table 3:** Questions included in the FJS questionnaire

	Are you aware of your artificial knee …
1	… in bed at night?
2	… when sitting on a chair for more than one hour?
3	… when you are walking for more than 15 min?
4	… when taking a bath/shower?
5	… when travelling in a car?
6	… when climbing stairs?
7	… when walking on uneven ground?
8	… when standing up from a low-sitting position?
9	… when standing for long periods of time?
10	… when doing housework or gardening?
11	… when taking a walk or hiking?
12	… when doing your favourite sport?

### Oxford knee score

The OKS scores of the matched groups are presented in Table 2. When comparing the matched groups, we found a statistically significant higher OKS score for the newer generation CR and for the MB-CR group when compared to their respectable previous generation CR TKA groups by 5 (*p* = 0.039) and 4 (*p* = 0.047) points respectively. The newer generation CR TKA achieved statistically significant higher scores in questions 1, 5, 9 and 11, while the MB-CR TKA achieved statistically higher scores in questions 4, 7, 9 and 11 [22], when compared to the previous generation CR TKA, respectively.

## Discussion

In recent years many efforts have been made in the development of new knee prosthetic designs with the aim of giving patients a more natural feeling knee during ADL. The present study was performed to determine if patients treated with a newer generation CR TKA or CR mobile bearing TKA design, would be able to achieve increased knee function and lower knee awareness during activities, when compared to patients treated with a widely used previous generation CR TKA. In this study the use of all three prosthetic designs showed good clinical results, comparable to the results found in previous studies of these prosthetic designs [11, 13, 17, 24].

The OKS scoring system was developed to evaluate knee pain and function of patients with osteoarthritis of the knee [22]. It has been widely used in earlier studies. A MCID of 4 to 5 points in OKS score has previously been identified [23]. However, the OKS score has some limitations. Because the OKS evaluates the patients’ ability to perform specific activities, the score is dependent on demographic elements such as BMI, age, gender and habitual activity levels [8]. The FJS system, however, was developed to assess knee awareness during ADL, hereby integrating a variety of variables such as pain, stiffness, function in activities of daily living, patients’ expectations and patients’ habitual activity levels [7]. Because the FJS scoring system evaluates the patients’ ability to forget the artificial joint during ADL (the ultimate goal in joint replacement surgery), this scoring system may be the optimal tool when evaluating the outcome after TKA. In an earlier study, Thienpont et al. found a high degree of differentiation when the FJS scoring system was used to evaluate differences in knee awareness of patients treated with unicompartmental knee joint replacement, patellofemoral joint replacement and TKA [8]. To our knowledge, the FJS scoring system has not been used to investigate differences in outcome between different TKA designs before this study.

Another limitation to the OKS scoring system is that it has shown to carry a considerable degree of ceiling effect [25]. In our study, we observed a ceiling effect (patients reaching a total score within 10 % of the maximum achievable score) of 31 % (99 of 316) for the OKS questionnaire vs. 12 % (37 of 316) for the FJS questionnaire, which is comparable to what has been documented in previous studies [7, 25–27]. We believe that this makes the FJS scoring system more suitable when investigating potentially small differences in performances of the knee of patients with good clinical results after TKA.

The Vanguard CR prosthesis (Biomet, Warsaw, Indiana) was developed with the intent of restoring high knee range of motion (ROM) along with improving patellar tracking and knee stability during all degrees of ROM in order to achieve a more natural knee function during ADL. In this study, we found that patients treated with this newer generation CR prosthesis had statistically significant higher FJS- and OKS scores when compared to patients treated with the previous generation CR prosthesis. The difference in OKS score of 5 points can be regarded as clinically relevant [23]. When looking at the individual questions of the FJS questionnaire (Table 3), we found that patients treated with the newer generation CR prosthesis achieved statistically significant higher scores in questions concerning knee awareness during night time, when taking a shower, travelling by car, climbing stairs and when rising from a low sitting position [7], all activities that patients are expected to be able to do in everyday life. Regarding the OKS questionnaire, we found statistically significant higher scores in questions concerning the patients’ perception of pain in everyday life, when rising from a sitting position, when performing usual work and the patients’ ability to do household shopping [22]. This taken into account, it seems that the development in prosthetic design of the newer generation CR TKA, including enhanced patellar tracking and increased stability during high degrees of flexion, could help the patient in achieving better knee function with a more natural feeling knee during ADL when compared to matched patients treated with the previous generation CR prosthesis.

The Vanguard ROCC prosthesis (Biomet, Warsaw, Indiana) is developed with a rotating tibial bearing and a deeper, longer trochlear groove in order to facilitate patellar tracking. The intent of this is to mimic the kinematics of the natural knee, hereby in theory increasing knee function, stability and patient satisfaction during all degrees of ROM. We found that the use of this MB-CR prosthesis was associated with statistically significant higher postoperative OKS-scores when compared to the previous generation CR prosthesis. The difference in OKS score of 4 points between the two prosthetic designs can be regarded as clinically relevant [23]. Patients treated with the MB-CR prosthesis achieved statistically higher scores in questions concerning the patients perception of pain when walking for longer periods of time and when performing usual work, and their ability to kneel down and do household shopping [22], activities that may demand high degrees of flexion. This could indicate that the use of a mobile bearing TKA with a femoral component that facilitates enhanced patellar tracking does result in a more natural functioning knee during full ROM in ADL. For FJS scores, we found that patients being treated with the MB-CR prosthesis achieved higher total scores than patients being treated with the previous generation CR prosthesis by 5 points. This difference in total FJS score, as well as the differences in the individual questions of the questionnaire, however, was not statistically significant.

There are some limitations to this study. First, a preoperative knee function score was not available. Second, we cannot rule out that this study might be subject to some degree of selection bias in that the surgeon may prefer one type of prosthetic design to a certain type of patient. The strength of this study, however, is the matching of patients between study groups in two stages regarding gender, age at surgery, time of surgery, K-L grade and pre- and postoperative knee alignment. Because of this matching procedure, we believe that our study groups are comparable regarding preoperative knee function. We cannot, however, rule out that differences in comorbidity load could be present between the study groups.

Although no statistically significant differences were found between the study groups after the matching procedure regarding demographical data, we found that patients in the previous generation CR TKA group were 4 and 3 years older than patients in the newer generation CR and MB-CR TKA groups, respectively. This difference in age could to some degree explain the difference in OKS score found in this study, as the OKS scoring system evaluates how well the knee performs during specific activities, which makes the OKS scoring system age dependent. The difference in age, however, cannot explain the difference in FJS score, as the FJS scoring system in theory is less dependent on the patients’ ability to perform specific activities of daily living and therefore is less dependent of patient age.

The follow-up period of 1 year for some patients in this study is relatively short and we can draw no conclusions about long-term knee function and awareness. Earlier studies, however, have revealed that knee function reaches a plateau beyond 1 year [28, 29] and therefore we believe that our results can be used as a good marker for long-term knee function and awareness.

## Conclusions

In conclusion, we performed a matched, case-control, cross-sectional survey based study on patients receiving a previous generation CR TKA (AGC), a newer generation CR TKA (Vanguard CR) or a mobile bearing CR TKA (Vanguard ROCC) at our institution between January 2010 and January 2013. We found that the patients receiving the newer generation CR TKA had higher FJS and OKS scores when compared to patients receiving a previous generation CR TKA. This could indicate that the use of this newer prosthetic design may facilitate less knee awareness potentially resulting in a more natural feeling during use of the TKA.

## Ethics statement

The Danish National Data Protection Agency approved this study (AHH-2014-010). Written informed consent to participate in the study has been obtained from participants. Approval by ethics committee is not required for retrospective questionnaire-based studies in Denmark (dnvk.dk).

## Availability of supporting data

Materials described in the manuscript, including all relevant raw data, will be freely available upon request to the corresponding author, to any scientist wishing to use them for non-commercial purposes, without breaching participant confidentiality.

## Abbreviations

ROM: range of movement
TKA: total knee arthroplasty
ADL: activities of daily living
PROM: patient reported outcome measures
FJS: forgotten joint score
OKS: Oxford knee score
KL: Kellgren-Lawrence
CR: cruciate retaining
MB: mobile bearing
AP: antero-posterior
MCID: minimal clinically important difference
